# Impact of an electronic antibiotic time-out best practice alert on antibiotic use and prescribing behavior in hospitalized patients

**DOI:** 10.1128/aac.01680-24

**Published:** 2025-05-20

**Authors:** Tyler Ackley, Joseph Kuti, Anastasia Bilinskaya, Kristin Linder, Casey Dempsey

**Affiliations:** 1 Department of Pharmacy Services, Hartford Healthcare, Hartford, Connecticut, USA; 2 Center of Anti-infective Research and Development, Hartford, Connecticut, USA; University of Pittsburgh School of Medicine, Pittsburgh, Pennsylvania, USA

**Keywords:** antimicrobial stewardship

## Abstract

Provider-directed electronic antibiotic time-outs (ATOs) are a stewardship strategy capable of efficient and widespread impact with relatively low perceived personnel effort. This is a multi-center, quasi-experimental, retrospective chart review of patients admitted receiving empiric antibiotics for >72 hours. An ATO alert was designed and embedded within the electronic medical record and set to fire between the hours of 07:00 and 16:30 daily. Seventy-two hours after a unique antibiotic order was entered, a best practice alert (BPA) would fire for the primary team—including the attending physician, residents, and advanced practice providers—as well as any infectious disease provider consulted at the time of ATO firing. This alert is triggered when entering or modifying orders as an active pop-up to the ordering prescriber. Prescribers were then prompted to assess the patient for potential antibiotic optimization—including discontinuation, de-escalation, and transition to oral therapies. A total of 800 patients receiving >72 hours of antibiotics were included for analysis. There was no statistically significant difference in the rate of antibiotic optimization when comparing the pre- and post-implementation cohort (54.5% vs 57.5%, *P* = 0.433); however, there was a numerically lower rate of antibiotic escalation in the post-cohort (9.5% vs 5.8%, *P* = 0.062). Duration of antibiotic therapy was longer in the post-implementation cohort (4.7 vs 5.0 days, *P* < 0.001). The implementation of an ATO BPA failed to improve the rates of antibiotic optimization.

## INTRODUCTION

In response to mounting antibiotic resistance and overuse, antimicrobial stewardship continues to serve as a major evolving initiative within healthcare institutions. Strategies to evaluate therapeutic regimens and optimize antimicrobial prescribing are numerous, with increasing attention focused on developing stewardship interventions capable of efficient and widespread impact. Antibiotic stewardship programs (ASPs) often employ active stewardship strategies, such as prospective audit and feedback, in conjunction with passive strategies, such as formulary restrictions and antibiotic time-outs (ATOs) (1, 2). The latter strategy encourages prescribers to self-govern antimicrobial use, prompting prescribers to re-evaluate therapy at key time points after a patient’s clinical status has improved or microbiology results have become available. For this reason, the Centers for Disease Control and Prevention recommend the addition of ATOs as a supplemental intervention to support optimal antibiotic use; however, there are no clear recommendations on the optimal timing or structure of ATO implementation (1, 2).

Studies evaluating the integration of ATOs into stewardship programs have been previously reported; however, their design, implementation, scope, and impact on antimicrobial therapy have been variable (3–6). Early iterations of ATOs described in the literature focused specifically on broad-spectrum agents, where a reduction of vancomycin use, but not piperacillin-tazobactam, was seen after ATO implementation (3). Despite this potential benefit, subsequent studies evaluating a larger breadth of antibiotics failed to see changes in overall antimicrobial days of therapy or hospital length of stay (LOS) (4, 5). Studies showing limited or neutral effects on antimicrobial therapy speculate that poor adoption of the ATO into prescriber workflows or potential alert fatigue may have compromised their potential benefit (5, 6). Based on these mixed findings, the optimal strategy surrounding ATO implementation and timing has not yet been established.

Despite the mixed results reported, an electronic ATO was implemented systemwide in our health system in September 2022 to promote therapy modification after early, empiric therapy selection. This ATO appears within the electronic medical record (EMR) as a best practice alert (BPA) for patients receiving an antibiotic for >72 hours and prompts prescribers to acknowledge the alert via a selection of clinical responses. The objective of this study was to evaluate changes in antibiotic use in hospitalized patients before vs after the system-wide implementation of an electronic ATO alert and describe prescribing behaviors associated with alert prompting.

## MATERIALS AND METHODS

This was a multi-center, quasi-experimental, retrospective study of prescriber interaction with an electronic ATO firing on patients admitted to one of seven hospitals within a large healthcare system who received empiric antibiotics for >72 hours. This study was approved by an internal institutional review board, and informed consent was waived. A pre-implementation control period (1 October 2021 to 31 October 2021) and a post-implementation period (1 October 2022 to 31 October 2022) were utilized to account for seasonality in antimicrobial prescribing. During the first week after the system-wide rollout, 10 antibiotics with the highest number of ATO alerts were selected for study inclusion. The top 10 antibiotics included the following agents: intravenous (IV) ampicillin-sulbactam, cefazolin, cefepime, ceftriaxone, meropenem, metronidazole, piperacillin-tazobactam, vancomycin, and oral (PO) metronidazole and doxycycline. These agents were further categorized into broad spectrum (cefepime, meropenem, piperacillin-tazobactam, and vancomycin), moderate spectrum (ampicillin-sulbactam and ceftriaxone), and narrow spectrum (cefazolin, IV/PO metronidazole, and PO doxycycline). Patients were eligible for inclusion if they were >18 years of age at the time of antimicrobial initiation and received an included top 10 antibiotic for >72 hours within the pre- or post-implementation period. Empiric antibiotics were defined as any new antibiotic order initiated in a patient previously off antibiotics for a minimum of 72 hours and started prior to the availability of microbiology results. Cultures obtained prior to or during the time of antimicrobial initiation were included if the results did not become available until after antibiotics were administered. Patients were included chronologically within the pre- and post-intervention cohort. If a patient was eligible for inclusion multiple times within the pre- or post-intervention timepoint, only the initial antibiotic orders were evaluated. Patients were excluded if they were receiving antibiotics for chronic suppressive therapy, surgical prophylaxis, or for a continuation of therapy for an infection diagnosed prior to hospitalization.

**Fig 1:**
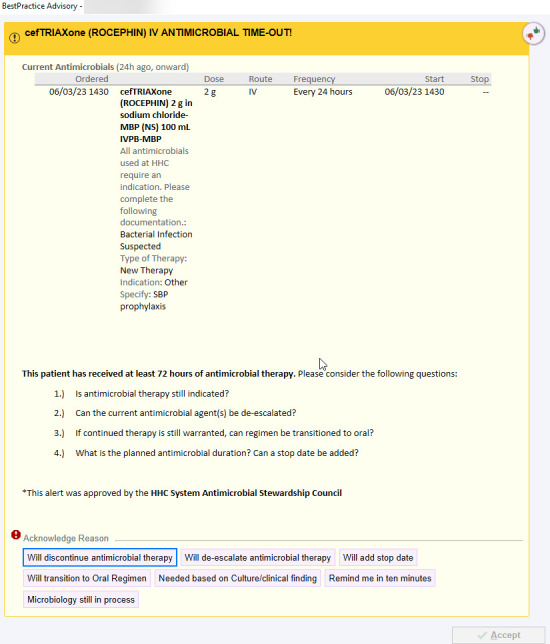
Example ATO interface visible to prescribers.

The ATO alert was designed and embedded within the EMR and set to fire between the hours of 07:00 and 16:30 every day. This alert fired for all users with prescribing authority upon entering the orders tab of the EMR on any patient chart with an antibiotic order at >72 hours of activity. The ATO fired for 50 unique antimicrobials, including IV and oral agents. The selected users included the primary team, including medical residents, fellows, and attending-level physicians, as well as advanced practice practitioners (APPs) and consultant clinicians. On ATO firing, prescribers were prompted to assess for antibiotic modification and were required to select from the following options including discontinuation, de-escalation, the addition of a stop date, and transition to oral therapies (Fig. 1). Additional selections were available to defer ATO re-firing based on culture results or clinical findings necessitating current antibiotics or if microbiology was still in process. The addition of the “remind me in 10 minutes” selection was added as a mechanism to temporarily bypass the ATO with the goal of giving providers ample time for chart review to assess if antibiotic modification was reasonable. Ten minutes was selected to provide reasonable time for patient review while also maximizing the possibility that the patient’s chart would still be in use. ATO responses are provider specific, and if the patient’s chart was accessed by a unique user, the ATO re-fires if no action was taken by the first provider that correlated with the response selected. A post-hoc assessment of the prescriber ATO responses was also evaluated to assess the adherence to the ATO selection. Adherence was defined as an ATO selection that corresponded to a clinical decision seen within 24 hours of ATO selection (e.g., an ATO selection of “will discontinue antibiotic therapy” was deemed adherent if antibiotics were then discontinued by any clinician within 24 hours). To assess adherence to the selected ATO response in patients for whom the ATO fired on >1 occasion, the first ATO response was documented and compared against the clinical changes made during the review period.

Throughout the ATO pre- and post-implementation period, the ASP staffing model and workflow remained consistent except for the adoption of a rapid diagnostic, PCR assay for bloodstream isolates in the post-implementation period. Implementation of a rapid diagnostic PCR occurred between the pre- and post-intervention cohort. These results, when available, were called a critical notification to the covering bedside nurse in real time. Prior to roll-out, prior ASP staffing duties were consistent among the seven sites and consisted of broad-spectrum audit and feedback led by three full-time ASP-trained pharmacists as well as a post-graduate year 2 infectious disease resident.

The primary endpoint was the rate of antibiotic modification as defined by the composite endpoint of discontinuation, de-escalation, the addition of a stop date, or the transition to oral therapy within 96 hours (24 hours after ATO firing). Selection of an ATO response alone was not sufficient, and modification of the antimicrobial orders outside of the ATO was required to achieve the primary endpoint. Dosing modifications as a reflection of changing renal function were not counted toward the primary endpoint. Additional secondary endpoints compared the rate of antimicrobial escalation, duration of therapy for included antibiotics, hospital LOS, infection-related LOS (defined as the time from antibiotic initiation to discharge), 30-day mortality, and rates of *Clostridioides difficile* infection (CDI) at 30 days. In patients who were discharged prior to 30 days after ATO firing, the absence of documentation surrounding readmission, mortality, or new CDI testing was presumed to be negative. A preset sample of 400 unique patients meeting inclusion criteria was included in each cohort. Patients were assessed for inclusion chronologically within the pre- and post-intervention cohort. Categorical variables were compared by a *χ*^2^ test or Fisher’s exact test. Continuous variables were compared using a Student’s *t*-test or Mann-Whitney *U* test. All findings were deemed statistically significant if they resulted in a *P*-value of <0.05. SigmaPlot v14 (Systat Software, San Jose, CA 2017) was used for all analyses.

## RESULTS

Eight hundred patients receiving >72 hours of the top 10 antibiotics were included for analysis, 400 patients in each cohort. Baseline characteristics between the pre- and post-intervention cohort were well balanced (Table 1), except for a statistically significant lower Charlson Comorbidity Index in the pre-intervention group (median, 4 [2–6] vs 5 [3–7]; *P* = 0.020). The most common indication for antibiotics was pneumonia (31.5% vs 34.3%), followed by urinary tract infections (15.0% vs 12.3%), intraabdominal infections (17.3% vs 14.5%), and skin and soft tissue infections (SSTIs; 11.0% vs 11.0%). These indications were similar between groups. More patients in the pre-intervention group received combination therapy with two of the evaluated antibiotic agents (117 [29.3%] vs 55 [13.8%]; *P* < 0.001). Similarly, a higher percentage of patients in the pre-intervention group received vancomycin compared with the post-intervention cohort (79 [15.3%] vs 26 [5.6%]; *P* < 0.001), whereas beta-lactam therapy was more common in the post-intervention group (358 [69.5%] vs 379 [81.9%]; *P* < 0.001). There were no differences in anti-pseudomonal beta-lactams between groups (112 [21.7%] vs 113 [24.4%], *P* = 0.363).

**TABLE 1:** Full bivariate analysis^*a*^

Baseline characteristics		Pre-cohort (*n* = 400)	Post-cohort (*n* = 400)	*P* value
Age {median (interquartile range [IQR]), years}		66 (55–78)	69 (58–79)	0.169
Female sex (no., [%])		195 (48.8%)	181 (45.3%)	0.357
Race (no., [%])	White	306 (76.5%)	308 (77.0%)	0.784
	African American	37 (9.3%)	37 (9.3%)	
	Other/unknown	57 (14.3%)	55 (13.8%)	
Charlson Comorbidity Index (median [IQR])		4 (2–6)	5 (3–7)	0.02
Intensive care unit admission (no., [%])		87 (21.8%)	78 (19.5%)	0.485
History of CDI (no., [%])		18 (4.5%)	22 (5.5%)	0.626
Infectious disease consult (no., [%])		151 (37.8%)	165 (41.3%)	0.347
Antibiotic evaluation				
Indication (no., %)	Pneumonia	126 (31.5%)	137 (34.3%)	0.452
	Urinary tract infection	60 (15.0%)	53 (12.3%)	0.542
	Intraabdominal infection	69 (17.3%)	58 (14.5%)	0.333
	SSTI	44 (11.0%)	44 (11.0%)	0.91
Dual antibiotics (no., [%])		117 (29.3%)	55 (13.8%)	**<0.001^*b*^**
Triple antibiotics (no., [%])		3 (0.8%)	4 (1.0%)	1
Beta-lactam (BL) (no., [%])		358 (69.5%)	379 (81.9%)	**<0.001**
Anti-pseudomonal (PsA) BL (no., [%])		112 (21.7%)	113 (24.4%)	0.363
VAN (no., [%])		79 (15.3%)	26 (5.6%)	**<0.001**
CRO or SAM (no., [%])		233 (45.2%)	246 (53.1%)	**0.016**

^*a*^ Baseline characteristics of included patients and antibiotics received during the pre- and post-ATO implementation cohort.

^*b*^ Bolded data is statistically significant.

There was no significant difference in the proportion of antibiotic modifications at 96 hours between the pre- and post-ATO cohorts (218 [54.5%] vs 230 [57.5%]; *P* = 0.433; Table 2). Despite no difference in the composite primary endpoint, there were fewer antibiotic stop dates utilized in the pre-ATO cohort (60 [15.0%] vs 125 [31.3%], *P* < 0.001). No other differences were seen between groups in other categories of antibiotic modification (e.g., discontinuation, de-escalation, or transition from IV to PO). Both LOS (median, 7.2 days [5.1–12.8] vs 8.2 days [5.9–14.7]; *P* = 0.003) and infection-related LOS (median, 6.5 days [4.7–10.3] vs 7.0 days [5.0–12.4]; *P* = 0.018) were higher in the post-intervention group. Additionally, the duration of included antibiotic therapy was higher in the post-intervention group (median, 4.7 days [3.8–6.3] vs 5.0 days [4.1–6.5]; *P* < 0.001). When the primary analysis was evaluated based on select antibiotic spectrum of activity, no statistically significant differences were observed.

**TABLE 2:** Primary and secondary outcomes^*a*^

	Pre-cohort (*n* = 400)	Post-cohort (*n* = 400)	*P* value
Antibiotic modification at 96 hours (no., [%])	218 (54.5%)	230 (57.5%)	0.433
Antibiotic discontinuation (no., [%])	98 (24.5%)	101 (25.3%)	0.870
Antibiotic de-escalation (no., [%])	51 (12.8%)	41 (10.3%)	0.319
Antibiotic stop date (no., [%])	60 (15.0%)	125 (31.3%)	**<0.001^*b*^**
Antibiotic IV to PO (no., [%])	70 (17.5%)	74 (18.5%)	0.782
Antibiotic escalation (no., [%])	38 (9.5%)	23 (5.8%)	0.062
CDI at 30 days (no., [%])	18 (4.5%)	22 (5.5%)	0.626
Mortality at 30 days (no., [%])	54 (13.5%)	47 (11.8%)	0.523
LOS {median (interquartile range [IQR]), days}	7.2 (5.1–12.8)	8.2 (5.9–14.7)	**0.003**
Infection-related LOS (median [IQR], days)	6.5 (4.7–10.3)	7.0 (5.0–12.4)	**0.018**
Antibiotic duration (median [IQR], days)	4.7 (3.8–6.3)	5.0 (4.1–6.5)	**<0.001**
Beta-lactam duration (median [IQR], days)	4.8 (3.8–6.2)	5.0 (4.0–6.3)	**0.025**
CRO duration (median [IQR], days)	4.6 (3.8–6.0)	4.9 (4.0–6.1)	0.065
CRO or SAM duration (median [IQR], days)	4.7 (3.8–6.0)	4.8 (4.0–6.1)	0.136
Anti-PsA duration (median [IQR], days)	4.8 (3.8–6.4)	5.6 (4.3–6.9)	**0.024**
VAN duration (median [IQR], days)	4.5 (3.7–6.7)	5.9 (4.7–7.8)	**0.012**
Antibiotic duration—pneumonia (median [IQR], days)	4.9 (3.9–6.5)	4.9 (4.0–6.0)	0.734
Antibiotic duration—urinary tract infection (median [IQR], days)	4.3 (3.6–5.7)	4.7 (4.1–5.9)	0.102
Antibiotic duration—intrabdominal infection (median [IQR], days)	4.3 (3.8–6.2)	4.7 (4.1–6.7)	**0.021**

^*a*^ Primary and secondary results of the pre- and post-ATO implementation cohorts.

^*b*^ Bolded data is statistically significant.

Information surrounding number of ATO alerts analyzed and hospital description is available in Table 3. Within the post-hoc assessment of the first prescriber ATO responses, the most selected ATO was “remind me in 10 minutes” (ATO 5), occurring 29% of the time (Table 4). The median number of ATO alerts per patient was 2 (interquartile range [IQR], 1–5). The median time viewing the ATO alert was 5 s (IQR 4–8 s). Overall, ATO selection adherence was low at 56%. When broken down by user role, attending-level physicians were more likely to adhere to their ATO selection when compared with APPs or resident-level physicians (62.7% vs 46.2% vs 48.5%; *P* = 0.007). Attending-level physicians were also more likely to select an ATO option corresponding to antibiotic modification (39.5% vs 12.3% vs 7.6%; *P* < 0.001) and less likely to defer the ATO re-firing for 10 minutes (14.5% vs 50% vs 45.5%; *P* < 0.001). There were no differences seen when ATO selection was broken down by prescriber specialty.

**TABLE 3:** ATO firing by site^*a*^

	Number of ATO alerts	Site description
Site 1	140	Tertiary Academic Center, 867 beds
Site 2	64	Academic Community Hospital, 446 beds
Site 3	56	Community Hospital, 213 beds
Site 4	50	Community Hospital, 473 beds
Site 5	49	Academic Community Hospital, 156 beds
Site 6	32	Community Hospital, 120 beds
Site 7	9	Community Hospital, 109 beds

^*a*^ ATO firing based on-site within the post-cohort.

**TABLE 4:** BPA assessment^*a*^

		*N* (%)		ATO adherence (%)	
ATO 0–6 (all)		400 (100)		56.0	
ATO 0–3 (discontinuation, de-escalation, the addition of a stop date, and transition to oral)		108 (27)		69.4	
ATO 4 (defer based on clinical status)		80 (20)		86.3	
ATO 5 (remind me in 10 minutes)		116 (29)		37.9	
ATO 6 (defer based on pending culture data)		96 (24)		37.5	
ATO adherence by user role (%)	Resident (*n* = 66)	Attending (*n* = 228)		APP^*b*^ (*n* = 106)	*P* value
	48.5	62.7		46.2	0.007
ATO adherence by specialty (%)	ID^*c*^ (*n* = 13)	IM^*d*^ (*n* = 205)	CC^*e*^ (*n* = 33)	Other (*n* = 89)	*P* value
	69.2	58.1	54.5	48.3	0.312
	Resident (*n* = 66)	Attending (*n* = 228)		APP^*b*^ (*n* = 106)	*P* value
ATO 0–3 by role (%)	7.6	39.5		12.3	**<0.001^*f*^**
ATO 5 by role (%)	45.5	14.5		50	**<0.001**

^*a*^ Post-hoc assessment of prescriber interaction with the ATO BPA.

^*b*^ Advanced Practice Practioner.

^*c*^ Infectious disease.

^*d*^ Internal medicine.

^*e*^ Critical care.

^*f*^ Bolded data is statistically significant.

## DISCUSSION

In this multi-center, quasi-experimental study, implementation of an electronic, prescriber-driven ATO was not found to be associated with an increased likelihood of antibiotic modification at 96 hours. Antibiotic modification remained low in both groups, nearing 50%, regardless of ATO implementation. These results are in line with some literature surrounding prescriber-driven ATOs that rely heavily on the incorporation of the ATO into daily workflow (5, 7). The available literature surrounding ATO implementation varies widely in terms of operationalization and incorporation into prescriber or pharmacist workflow. Other studies have employed more active strategies, such as utilization of a structured discussion on antibiotic therapy during rounds or employing prescribers to post a note in the EMR, which differ greatly from the comparatively passive ATO BPA used here (4, 6). These active strategies are more likely to engage prescribers; however, they may take more time away from other aspects of patient care. Active review of ATO responses by ASPs—potentially via tailored audit and feedback—may strategically reinforce the ATO message and, as such, improve the rates of antibiotic modification. Unlike Wolfe et al., who similarly utilized an ATO BPA strategy, our study assessed the rate of antibiotic modification in all patients, not just those with available culture results (8). Oftentimes, hospitalized patients will not have culture data to drive de-escalation, and de-escalation must be driven by clinical stability. Additionally, Wolfe et al. focused on broad-spectrum antibiotic therapy specifically, whereas we included our system-wide top 10 agents, many of which would be considered narrow spectrum. These central differences may explain the improvement in de-escalation rates seen in their post-implementation cohort (35.1% vs 55.0%, *P* = 0.0095) when compared to our neutral results (8).

An additional challenge that may have hindered antibiotic modification may stem from the interface of the ATO itself. Our ATO required the provider to select an ATO response and then modify the orders manually. Given the multiple selection options available, especially those including de-escalation and transition to oral antibiotics which are diverse and patient/disease-specific in nature, a simplified and concise ATO was designed without the ability to modify orders from within the ATO interface. Future attempts at ATO development may benefit from including potential opportunities for de-escalation or oral transition within the BPA, or designing disease-specific ATOs which can be more directed and narrow in scope. Further potential consequences of this ATO build include worsening alert fatigue given the active strategy employed. Contrasting a passive banner alert, this ATO required review by prescribers before continuing with various tasks within the patient chart. While the number of ATOs fired per patient was low (2, IQR 1–5) and the median time viewing the alert was short (5 s, IQR 4–8 s), the combined impact of antibiotic and non-antibiotic disruptive alerts within the EMR may negatively impact prescriber workflow and further burnout (9). Prescribers were given the opportunity to provide feedback on the ATO within the alert interface; however, this feedback was low in number (*n* = 3), limiting further analysis.

The decision to analyze ATO firing in response to a pre-specified list of the top 10 most utilized agents, in lieu of more traditionally broad-spectrum antibiotics, was chosen to better assess the ATO impact across various spectra of activity. Given that many ASPs provide audit and feedback primarily on broad-spectrum agents, this ATO would fire on antimicrobials across both broad, moderate, and narrow spectrum of activity and, therefore, include agents that are not routinely audited. In addition, narrow-spectrum agent prescribing could potentially benefit from tailoring durations of therapy or transitioning to an oral agent. Ultimately, when analyzed for the primary endpoint, there were no differences seen in the rates of antibiotic modification across the antibiotic spectrum categories: broad (53.5% [77/144] vs 47.1% [57/121], *P* = 0.302), moderate (55.9% [127/227] vs 62.3% [152/244], *P* = 0.161), or narrow (48.3% [14/29] vs 60.0% [21/35], *P* = 0.348). When narrow-spectrum agents were excluded, this analysis remained neutral (55.0% [204/371] vs 57.3% [211/368], *P* = 0.5197), suggesting that the negative results seen by the ATO were not specifically driven by low-spectrum agents.

When focusing on antimicrobial utilization, there were some key differences observed between the pre- and post-intervention cohorts. Significantly fewer patients in the post-intervention cohort received vancomycin (79 [15.3%] vs 26 [5.6%]; *P* < 0.001). Our findings mirror the results seen with other ATO studies. Graber et al. identified a significantly higher rate of vancomycin discontinuation between days 3 and 5 after ATO implementation (64% [93/145] vs 48% [96/199]; *P* = 0.004); however, through provider self-stewardship, there was a low—yet increased—rate of inappropriate vancomycin continuations (5% vs 0%; *P* = 0.002) (3). It is important to note, however, that appropriateness was determined by two infectious disease providers, whereas in their pre-ATO cohort, vancomycin use extending past 3 days required ID approval. The reasoning for the decrease in vancomycin use seen in our post-intervention cohort may be driven in part by ATO design, in which the ATO would fire if an order was active for >72 hours. Contrasting Graber et al., within our institution, vancomycin can be ordered for empiric and definitive therapy without ID consultation. Additionally, dosing is pharmacist driven, where dosing is dependent on clinical status and changes in serum creatinine or urine output. As such, patients with an unstable renal function requiring renally dosed medications—including vancomycin—may have been underrepresented in the post-intervention cohort if an order dose or frequency was modified prior to the 72-hour alert firing. The addition of rapid diagnostic technologies for bloodstream isolates in the post-intervention period may have further reduced the empiric use of vancomycin, as the identification of a Gram-negative pathogen would routinely allow for early vancomycin discontinuation. Similarly, these challenges may have led to the higher proportion of patients on dual antibiotic therapy seen in the pre-intervention group.

The ability to safely de-escalate or discontinue antibiotics is often associated with improvement in clinical stability and readiness for infectious-disease-related discharge. Despite similar rates of the antibiotic modification seen at 96 hours, we saw an increase in overall length of stay between our pre- and post-intervention cohort (7.2 days [IQR 5.1–12.8] vs 8.2 days [IQR 5.9–14.7]; *P* = 0.003). To help account for potential non-infectious etiologies driving the statistically significant difference, we also calculated LOS from antibiotic initiation, which was also increased (6.5 days [IQR 4.7–10.3] vs 7.0 days [5.0–12.4]; *P* = 0.018), although this difference was numerically lower than when overall LOS was assessed. These differences may be in part driven by the subtle but statistically increased Charlson Comorbidity Index score in the post-intervention cohort (4 [IQR 2–6] vs 5 [IQR 3–7]; *P* = 0.020) that potentially led to challenges with discharge placement due to the presence of select comorbidities. Other studies, namely Taylor et al., similarly noted an increase in both overall LOS and antibiotic LOS after ATO implementation (8 days [IQR 6–15] vs 11 days [7–21]; *P* = 0.02) and (8 days [IQR 6–11] vs 10 days [IQR 7–16]; *P* = 0.0006), respectively (6). Unlike Taylor et al., however, we did not exclude patients in which an ATO fired within 48 hours, which may have in part driven the larger numerical differences seen in their study. Through the selection of a 72-hour timepoint, our study may have selected patients who were more likely to be infected as deemed by the treating clinician team. The increase in LOS seen in our study may have played a role in the increase in calculated antibiotic duration. Given that the rate of antibiotic modification was partly driven by transitions to oral agents, a change that often occurs to help facilitate discharge, the longer LOS may have, in part, provided an opportunity for prescribers to continue intravenous therapies.

To the authors’ knowledge, this is the first ATO study to assess the differences in ATO response by prescriber specialty and role within the care team. The stark differences seen between resident-level physicians and APPs vs attending-level physicians, oftentimes hospitalists, showcase that the latter group may be an ideal target of ATO BPAs. Selection differences, namely a higher utilization of the “remind me in 10 minutes” selection, may be driven by the academic nature of our healthcare system, where resident-level physicians or APPs may be reviewing patient charts prior to rounding as a larger team. We were unable to identify differences in ATO selection or adherence when prescribers were separated by specialty. Numerically, ID prescribers had the highest ATO selection adherence; however, the low numbers of ID prescribers included may be driven by ATO design. Oftentimes, as a consulting specialist, ID prescribers within our system may discuss treatment recommendations with the primary team in lieu of placing orders directly. Given that the ATO fired when placing new orders, this may have limited ID prescriber ATO assessments, despite the high percentage of ID consultations ordered at the time of ATO firing included in our study. Further work leveraging ID prescriber involvement may have improved the impact of this ATO BPA given the preliminary data seen here. Prior to our study, the previous largest ATO BPA study was Wolfe et al., corresponding to 200 patients in total (8). Given the multicenter nature of our health system, we were able to include fourfold more patients, providing a more thorough investigation of the impact of a prescriber-driven ATO.

This study has several limitations given its focus on overall antimicrobial use without evaluation of the appropriateness of antimicrobial therapy. The decision to focus on antimicrobial modification and utilization was driven by the retrospective nature of the study and the comparatively limited clinical understanding that may come from chart review alone. As such, it was felt by the authors that an attempt to evaluate the clinical appropriateness of antibiotic therapy may introduce unnecessary bias. The higher median Charlson Comorbidity Index seen in the post-cohort may have neutralized any potential positive impact in antibiotic modification attributed to ATO implementation. These differences may also help explain the longer duration of antibiotic therapy and LOS. Similarly, this study focuses on a group of 10 select antibiotics, determined by specific system-wide utilization data. As such, modifications to antibiotic classes such as macrolides or fluoroquinolones, which may represent a larger component of total antibiotic therapy at other centers, were not evaluated. Additionally, a 24-hour evaluation period was used to assess antibiotic changes post-ATO firing to ensure all prescribers had ample time to assess antibiotic therapy. Other studies assessing ATO implementation have used evaluation periods of 6–24 hours (5, 7). Given that the ATO was designed to fire until 16:30, reducing the period for evaluation to 6 or 12 hours would have potentially limited a rounding team’s ability to evaluate and make changes to antibiotic therapy. Increasing the evaluation period to 24 hours would allow adequate time for non-attending-level prescribers to discuss antibiotic changes without being unnecessarily penalized for bypassing the ATO.

Optimization of antimicrobial therapy is a fundamental goal of antimicrobial stewardship efforts; however, many ASPs are limited in their ability to review all antimicrobials ordered due to staffing challenges and overall workload. The Centers for Disease Control and Prevention recommend the use of ATOs in combination with traditional prospective audit and feedback to help reduce unnecessarily broad antibiotic therapy. Despite these recommendations, these data alone may not support the adoption of an ATO-based stewardship strategy into a healthcare system with a developed ASP; however, it provides useful insight into the challenges seen with prescriber-directed self-stewardship. Further work, including ATO redesign, prescriber education, and ID provider support, may be necessary to optimize the impact of similar intervention strategies on antibiotic modification.

In response to the negative results seen in this analysis, our institution has made major modifications to the ATO. Given the large percentage (32.9%) of pneumonia seen in this cohort, a community-acquired pneumonia BPA was developed, including options within the ATO for discontinuation and oral transition. We hope that by moving toward a more focused group of antibiotics and allowing ordering directly through the BPA, providers will feel more empowered in self-stewardship. Further work developing alternative disease-state BPAs is ongoing. In conclusion, the addition of an electronic ATO embedded within the EMR was not associated with improvements in antibiotic modification at 96 hours. Based on our findings, passive strategies to improve antibiotic utilization are not a replacement for active review by dedicated ASP staff, and further work is warranted to optimize ATO alerts to leverage appropriate antimicrobial stewardship best practices.
